# OCT – An Insight into Retinal Disorders

**DOI:** 10.4103/0974-9233.51982

**Published:** 2008

**Authors:** Ravi Keshavamurthy, Sandeep Grover

Optical coherence tomography (OCT) has emerged as an effective imaging tool in the evaluation and management of retinal disorders. Based on the reflectance of the near-infrared light rays from different layers of retina, retinal pigment epithelium (RPE) and choroid, it creates cross-sectional images of these layers. The non-invasive nature of the test and the ability to image posterior segment structures in vivo with resolution approaching that of near-histological sections has made OCT particularly useful in the diagnosis, prognosis and management of retinal diseases.

Since its introduction in the late 1990's for clinical application in the imaging of retinal disorders, OCT has shown major improvements in technology. The initial versions (Stratus OCT), based on optical time-domain technology, use several A-scans through retina/RPE/choroid complex to ‘construct’ its image. The axial resolution (which depends on the wavelength and bandwidth of the instrument's light source) of Stratus OCT is 10 microns. The signals generated from the nerve fiber layer and the junction between the outer and the inner segments of the photoreceptors have maximum intensity and the distance between the two layers is used to measure the retinal thickness. In fact, this technology helps to identify subtle changes in these layers that can be missed on clinical examination, fluorescein angiography or ultrasound imaging. However, conventional time-domain OCT (TD-OCT) has several limitations. Since there is a time delay involved during the axial translation of the reference mirror in the instrument, the number of A-scans acquired is limited, resulting in a B-scan with poor resolution. Another related problem is the lack of ‘registration’, a point-to-point correlation between an OCT B-scan and fundus features. A critical limitation related to the slow-speed of TD-OCT is poor sampling density – a large amount of data is interpolated by sampling only a fraction of the mapped area.

Advances in OCT technology in the past few years have increased the resolution of images. Spectral-domain OCT (SD-OCT), also known as Fourier-domain OCT, increases the resolution of the image by two ways - firstly, by increasing the bandwidth of the incident light rays and secondly, by increasing the acquisition time of scans. SD-OCT acquires images 50 -100 times faster than TD-OCT. To further enhance resolution, multiple spectral-domain scans of the same location are imaged, which then are post processed by the instrument to reduce the noise. Hence, a large number of scans are acquired in the least amount time, enhancing the resolution of the OCT. Among the commercially available SD-OCT's, Spectralis (Heidelberg Engineering, Vista, CA / Germany) scans the highest number of A-scans (40,000 A-scans/sec, which is 200 times more than Stratus OCT which scans about 200 A-scans/sec). Another important distinction is that Spectralis OCT measures retinal thickness between the nerve fiber layer and the RPE-Bruch's membrane complex (unlike Stratus, which measures only up to the junction of inner and outer segments of photoreceptor cells). In addition, Spectralis is the only SD-OCT to incorporate the Trutrack™ technology, which significantly reduces image corruption due to motion artifacts and provides the opportunity to correlate quantitatively the same areas of retinal pathology at sequential time points. To summarize, Spectralis OCT is faster, has better resolution, more accurate and better reproducibility.

OCT is an important adjunct to other investigative modalities like fundus fluorescein angiography and ultrasound in the diagnosis and management of various retinal disorders. This technology has been most widely used in detection and monitoring of macular edema, irrespective of the cause. So much so, TD-OCT has become an essential test modality in clinical trials investigating the efficacy of various forms of treatment for diabetic macular edema. It not only provides the retinal thickness in macular edema but also provides volumetric analyses of various segments of retina. It has enabled ophthalmologists to diagnose and manage other retinal conditions such as age-related macular degeneration (AMD), central serous retinopathy and other inherited retinal diseases.

With the advent of SD-OCT, however, it has revolutionized our understanding of the pathophysiology of various disorders like macular degeneration. It has enhanced our knowledge of these diseases at a tissue level, providing us with in vivo near-histological images of these retinal structures. The improved imaging of the outer retinal surface has resulted in a more detailed study of the pathology involving the RPE and the outer segment of the photoreceptors in cases of AMD. Disruption of the retinal architecture in macular edema and its response to treatment is far better visualized with SD-OCT. Better delineation at the vitreoretinal interface by SD-OCT has enhanced the identification of epiretinal membranes, posterior vitreous detachment, vitreomacular traction and macular holes.

Ophthalmology is at the forefront of benefiting from such technological advances due to the easy accessibility in observing the disease process and its reversal with effective treatment. OCT has ushered in a new era and is an excellent example of application of technology to the understanding of diseases as ophthalmologic testing moves from a tissue to a cellular level.

